# Communal narcissism and internet altruistic behavior of Chinese college student: A moderated mediating effect

**DOI:** 10.1371/journal.pone.0342680

**Published:** 2026-02-24

**Authors:** Zimi Li, Xiangkui Zhang

**Affiliations:** 1 College of Humanities and Arts, Shenyang University of Chemical Technology, Shenyang City, Liaoning Province, China; 2 Northeast Normal University, Changchun City, Jilin Province, China; University of Padova, ITALY

## Abstract

This research aimed to examine the relations among communal narcissism, Internet impression management, self-esteem and Internet altruistic behavior. This study measured 700 university students from 3 universities in the Northeast using the Narcissistic Personality Inventory, the Internet Impression Management Scale, the Self-Esteem Scale, and the Internet altruistic Behavior Scale, with 675 effective participants. The results of the study showed that the direct effect of communal narcissism on university students’ Internet altruistic behavior was significant. After adding Internet impression management as a mediating variable, the effect of communal narcissism on university students’ online prosocial behavior remained significant. At the same time, self-esteem played a moderating role in the relationship between communal narcissism and Internet impression management.

## 1. Introduction

With the rapid development of the Internet, the network has become an important part of people’s lives, and people’s psychology and behavior on the Internet have naturally become a hot topic of researchers’ attention. According to the “52nd China Internet Development Status Statistical Report, “the proportion of Internet users aged 20–29 is 14.5% [1]. College students, as the main users of the Internet, and because the values of college students are in the formative stage, exploring the online behavior of college students helps to cultivate their correct online behavior views [2].

Internet altruistic behavior refers to voluntary and intentional actions taken by individuals online to promote the well-being of others or society, such as helping, guiding, sharing, and reminding, typically without expectation of direct personal gain [3]. Common manifestations include providing assistance, sharing knowledge, spreading positive information [4],gaining social recognition, establishing a favorable online image, and fulfilling personal achievement needs [5]. It is important to note that this definition primarily focuses on observable, other-benefiting online behaviors themselves, rather than making moral assumptions about underlying motivations. Theoretically, altruistic behaviors can be categorized into endogenous altruism (driven by genuine concern for others’ welfare) and instrumental prosocial behavior (motivated by goals such as self-enhancement).

According to Uses and Gratifications Theory, individuals with high self-fulfillment needs often engage more actively in self-presentation on online platforms [6,7,8]. Communal narcissism is a personality trait characterized by a tendency to exaggerate one’s positive qualities in interpersonal or moral contexts, with a strong emphasis on shaping and maintaining a public image through visible, socially desirable behaviors [9]. Individuals high in communal narcissism frequently display marked altruistic-oriented behaviors; however, their underlying motivations are likely geared more toward gaining social recognition and sustaining a positive self-image, rather than arising purely from concern for others’ welfare [10,11]. Thus, while the behaviors of communal narcissists may appear altruistic on the surface, their motivations are often instrumental, aimed at obtaining validation, attention, or self-affirmation through self-enhancement, representing a form of strategic prosocial behavior.Therefore,this study aims to examine the impact of communal narcissism on college student Internet altruistic behavior.

According to impression management theory [12], individuals have a proactive need to manage their personal image in order to maintain a positive impression of themselves in the eyes of others. Previous research has found that individuals with higher levels of narcissism are more concerned about their image in the eyes of others, and they will try various ways to protect their positive image [11]. In the online environment, good Internet impression management can help college students gain more attention and recognition in the virtual space, so individuals with high levels of communal narcissism will work harder to manage their Internet impression, and good Internet impression management can also promote individuals to show more prosocial behavior and more altruistic behavior online.

Furthermore, according to social sociometer theory [13], self-esteem is the manifestation of an individual’s positive self-concept, and good self-esteem helps individuals to exhibit more prosocial behavior. The level of self-esteem also affects the behavior individuals adopt when facing others, and individuals with different levels of self-esteem have different levels of self-presentation. A study involving Polish and British university students found that there are differences in the relationship between communal narcissism and self-esteem in different cultures [14]. Compared to individualistic cultures, the relationship between the core values of mutualism and self-esteem is more closely related in collectivist cultures. Individuals with higher self-esteem are more concerned about their image and hope to leave a good impression on others [15].

In summary, this study investigates the mediating role of Internet impression management in the relationship between communal narcissism and Internet altruistic behaviors among college students in the context of Chinese collectivist culture, as well as the moderating role of self-esteem in the relationship between communal narcissism and Internet impression management.

### 1.1 Communal narcissism and internet altruistic behavior

The concept of Internet altruistic behavior originates from altruistic behavior [16,17], Pen & Fan,2005; [18], which includes several characteristics: (1) Beneficial to others; (2) Behavior is voluntary; (3) Behavior is intentional; (4) Altruistic purpose; (5) Do not expect any spiritual or material rewards [19]. Internet altruistic behavior uses the internet to proactively post valuable information to alert others, as well as to respond positively or repost when seeing others seeking help [16].

According to the uses and gratification theory [8], an individual’s use of social networks is for the purpose of satisfying specific psychological needs. The use of social networks meets the individual’s need for positive self-presentation, seeking a sense of belonging, obtaining social support, and engaging in positive self-construction [8]. The internet breaks the boundaries of space and time, allowing people to seek social identity and establish online social relationships.

The anonymity of the internet makes it easier for individuals to engage in altruistic behavior [20]. Individuals maintain a relatively positive self-image through various regulatory processes involving the self, affect, and field, which is a crucial motivation for individuals to seek self-enhancement from the social environment [21]. Communal Narcissism refers to the exaggeration of positive traits in interpersonal interactions or in behavioral and moral judgments [9]. Previous studies have found that communal narcissism is closely related to Internet altruistic behavior in college students [22–25], Social networking tools provide a platform for individuals to receive appreciation and recognition from others (likes, comments, and shares). Communal narcissists seek attention, approval, and recognition from others through Internet altruistic behavior to satisfy their own need for high self-esteem and self-worth [15]. College students are at an important stage in the formation of their worldviews and values. As the main user group of online media, they exhibit more online behavior and are more willing to showcase themselves through the internet to gain recognition and ensure personal satisfaction. At the same time, in online interpersonal interactions, they often tend to exaggerate their positive performance in altruistic behavior to attract more attention and achieve a positive self-concept [22,25–27].

In conclusion, it is necessary to study the relationship between public narcissism and online altruistic behavior to reveal the intrinsic motivation behind online altruistic behavior. Based on this, this study suggests that there is a significant correlation between public narcissism and online altruistic behavior in college students, with higher levels of public narcissism being associated with more online altruistic behavior.

### 1.2 The mediating role of Internet impression management

Internet impression management refers to the behavior of individuals presenting themselves online, managing their own image by displaying relevant texts, images, links, and other content [27]. In interpersonal interactions, this practice plays a crucial social-adaptive role, enabling individuals to achieve more favorable social outcomes. Unlike face-to-face interactions, online environments offer anonymity and asynchronicity, allowing individuals to exert greater control over the content and timing of their self-presentation, thus more effectively projecting an idealized self [28,29]. Compared to real-life social activities, the non-immediacy of online social interaction can allow individuals to better control the timing and manner of their online communication. It also enables them to present carefully curated content on social media that reflects an idealized state of self-management [29–32]. The virtual and delayed nature of the internet gives individuals a sense of control and security, making them more willing to choose online self-presentation as a way to communicate and build relationships with others [33,34], This allows others to form a more positive and morally upright impression of oneself [80], understand one’s intentions [35], and enhance public recognition and acceptance of one’s image through interpersonal communication [36].

Active Internet self-presentation is often driven by the desire to meet psychological needs and engage in self-display [37]. Through impression management, individuals can shape interpersonal relationships, influence perceptions, and leave a favorable impression [33,38]. Research shows a strong link between university students’ Internet impression management skills and their Internet altruistic behavior: better impression management is generally associated with more frequent altruistic acts online [39,40]. By using platforms like WeChat Moments, TikTok, and Facebook for self-presentation, emotional sharing, and social network expansion, students gain satisfaction and a sense of social connection from feedback [41]. Positive self-presentation also facilitates emotional release, boosts self-esteem and self-awareness, and promotes self-development [42,43]. This may further encourage prosocial behavior, helping to establish and maintain a positive public image.

According to the uses and gratifications theory, people engage with social media to fulfill psychological needs [44,45]. Communal narcissists display more altruistic behaviors in order to gain attention, approval, and recognition from others. However, these behaviors often involve exaggerated self-presentation, which can lead to questions about their sincerity and risk negative evaluations [23]. To mitigate such negative perceptions, communal narcissists deliberately use impression management strategies to align their Internet altruistic acts with social expectations, thereby preserving or enhancing their self-image. Research indicates that effective impression management skills enable highly narcissistic individuals to more successfully seek external attention on social media [46], project a positive self-image [47], and gain recognition through open expression and interaction[48]. Through this strategic self-presentation—by attending to and responding to others’ feedback—communal narcissists demonstrate increased Internet altruistic behavior [49].

Based on the above theory and research, this study believes that Internet impression management plays a mediating role in communal narcissism and college students’ Internet altruistic behavior.

### 1.3 The moderation of self-esteem

If communal narcissism influences college students’ Internet altruistic behavior through internet impression management, are there other moderating factors that could further enrich our understanding of this relationship? This study focuses on the important role of self-esteem in this process.

Self-esteem refers to an individual’s overall evaluation of their own worth and competence, which functions as an internal sociometer that monitors and responds to one’s relational value in social contexts [50]. According to sociometer theory, self-esteem serves as a regulator of interpersonal relationships [51], shaping how individuals perceive social feedback and adjust their subsequent behaviors [52,53]. Previous research has shown a strong positive correlation between self-esteem and communal narcissism [54]. While the two constructs are conceptually and functionally distinct—communal narcissism reflects motivated self-enhancement in the prosocial domain, whereas self-esteem represents a more global, affect-laden self-evaluation—individuals with higher levels of narcissism also tend to report higher levels of self-esteem and a stronger sense of superiority, enabling them to maintain a positive self-view and favorable self-evaluations [55].Individuals with high self-esteem possess greater psychological resources and are more resilient to ego threats [56]. When communal narcissistic motives are activated (e.g., the desire to be perceived as exceptionally helpful or moral), those with higher self-esteem may feel more secure in translating these motives into observable Internet actions [57]. In contrast, those with lower self-esteem may avoid such behaviors due to fear of failure or negative evaluation.

Moreover, self-esteem shapes the strategic expression of prosociality. Whereas communal narcissism predicts a desire for prosocial recognition, self-esteem influences whether this desire is expressed through sustained impression management and altruistic acts or remains merely a self-enhancing fantasy [39,58].Individuals with high communal narcissism are particularly motivated to engage in behaviors that bolster their prosocial image, such as Internet altruism [15,14]) and impression management [56,59,60]. However, the extent to which they actually enact these behaviors may depend on their baseline level of self-esteem.According to the psychodynamic mask model [61], narcissism and self-esteem have a certain dynamic relationship [62]. For instance, some studies report a positive correlation between self-esteem and impression motivation [39], whereas others find no significant link between self-esteem and Internet impression management [63]. Such discrepancies underscore the complex, dynamic interplay between narcissism and self-esteem, as conceptualized in psychodynamic models such as the mask model [61]. These inconsistencies highlight the need to systematically examine how self-esteem moderates the relationship between communal narcissism and Internet impression management. Therefore, it is necessary to study the role of self-esteem in the relationship between communal narcissism and Internet impression management.

Based on the above research, this study suggests that self-esteem plays a moderating role in communal narcissism and college students’ Internet impression management.Self-esteem has a moderating effect on communal narcissism and college students Internet altruistic behavior.

### 1.4 The present study

This study explores the relationship between communal narcissism, Internet impression management, self-esteem and college students Internet altruistic behavior. Research can help reveal the underlying mechanisms of college students Internet altruistic behavior and provide more effective intervention strategies for college students Internet altruistic behavior. Based on previous theoretical and empirical research, we propose the following assumptions:

H1: Communal narcissism will be positively associated with college students Internet altruistic behavior.

H2: Internet impression management mediates between communal narcissism and college students Internet altruistic behavior.

**Fig 1:**
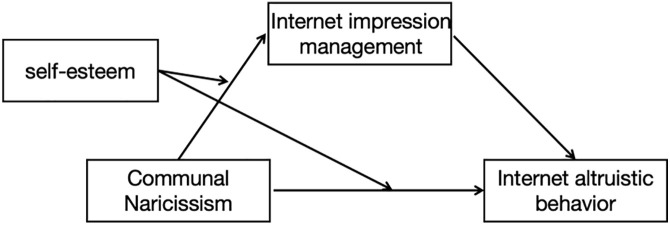
The hypothesis model of present study.

H3: Self-esteem has a moderating effect on communal narcissism and college students Internet Impression management. Self-esteem has a moderating effect on communal narcissism and college students Internet altruistic behavior Fig 1.

## 2 Method

### 2.1 Procedure and participants

Data were collected in June 2023 via the online survey platform “Questionnaire Star.” The platform required respondents to complete all items before submission, thereby ensuring a dataset without missing values. Prior to participation, all participants reviewed and agreed to an online informed consent form that outlined the study’s purpose, procedures, and confidentiality measures. To ensure data quality, attention-check items (e.g., “Please select ‘4’”) were embedded throughout the survey. Responses that failed these checks were considered invalid and excluded from analysis. The study was approved by the Academic Ethics Committee of the School of Psychology at Northeast Normal University.

A total of 700 college students from three universities in Northeast China (e.g., Liaoning Province) initially participated. After removing respondents who failed the attention checks, the final sample consisted of 675 students. Among them, 419 (62.1%) were male and 256 (37.9%) were female. The sample included 138 freshmen (M = 19.09, SD = 0.37), 164 sophomores (M = 20.07, SD = 0.25), 172 juniors (M = 21.05, SD = 0.24), 95 seniors (M = 22.17, SD = 0.43), and 106 graduate students or above (M = 24.03, SD = 0.71).

### 2.3 Materials

#### 2.3.1 Communal narcissism scale.

The Communal Narcissism Scale, compiled by [15,64], was used in this study. Some modifications were made to the original English version of the scale, resulting in a total of 16 items scored on a scale ranging from “strongly disagree” (1) to “strongly agree” (7). The Cronbach’s alpha coefficient for the scale in this study was 0.93, and the Cronbach’s alpha coefficients for the current and future Communal Narcissism Scales were 0.86 and 0.90, respectively.

#### 2.3.2 Internet altruistic behavio*r.*

The Internet Altruistic Behavior Scale was developed by [3] and is based on the “Internet Altruistic Behavior Scale” for college students. The questionnaire consists of 26 items, scored on a 4-point Likert scale. The higher the individual’s score, the more internet altruistic behaviors they exhibit. In this study, the reliability coefficient of the scale was 0.96.

#### 2.3.3 Internet impression management.

The “College Students’ Internet Impression Management Tendency Questionnaire” compiled by Ma Lina was used [65], consists of 20 items divided into 2 factors: other-oriented impression management tendency and self-oriented impression management tendency. Among them, there are 11 items for other-oriented impression management and 9 items for self-oriented impression management. The internal consistency coefficient of the total scale is 0.846, and the internal consistency coefficient of the two dimensions is 0.843 and 0.731 respectively. The scale uses Likert point scoring.

##### 2.3.4 Self-esteem.

The [66,67] was used, consisting of 10 items scored on a point scale ranging from “completely disagree” to “completely agree”, with one reverse-scored item. For example, “I feel that I am a person of worth, at least on an equal plane with others.” The overall Cronbach’s alpha coefficient for the scale in this study was 0.83.

### 2.4 Data analysis

First, descriptive statistics and Pearson correlation analysis were conducted using SPSS 26.0. To examine common method bias, Harman’s single-factor test was performed by including all items in an unrotated principal component analysis. The results showed that the first factor accounted for less than 40% of the total variance, indicating that common method bias was not a serious concern . Subsequently, mediation and moderation analyses were conducted using the PROCESS macro (Version 8.3) developed by Hayes . Model 4 was used to examine the mediating role of online impression management in the relationship between public narcissism and online altruistic behavior. Model 8 was applied to test the moderated mediation model [68], specifically assessing the moderating effect of self-esteem on the relationship between public narcissism and online altruistic behavior, as well as its moderating effect on the relationship between public narcissism and online impression management. Prior to analysis, all variables were standardized. Furthermore, the bootstrap method with 5,000 resamples was employed to calculate the 95% confidence interval (CI) for the indirect effects. An indirect effect was considered statistically significant if the 95% CI did not include zero.

## 3 Results

### 3.1 Common method bias

In order to avoid common method bias, this study implemented controls in the procedures, such as anonymizing certain survey items and using reverse wording. Furthermore, to further enhance scientific rigor, the study employed the Harman single-factor test to examine common method bias. The results showed that there were six factors with an eigenvalue greater than 1, and the first factor explained only 25.57% of the variance, which is below the critical standard of 40%. This indicates that there is no significant common method bias in the data of this study.

### 3.2 Descriptive statistics and correlation analysis

Table 1 presents the means and standard deviations of all the study variables and the results of the correlation analyses of the study variables. An independent samples t-test was conducted on gender, and the results indicated a significant gender difference in Internet altruism behaviors(t = 2.19, p < 0.05). Therefore, gender was included as a control variable in subsequent tests of moderating variables.Communal narcissism positively correlated with Internet altruistic behavior, Internet impression management and self-esteem. Communal narcissism, Internet altruistic behavior, Internet impression management, and self-esteem are all significantly positively correlated with each other. In addition, the relationship between gender and communal narcissism is not significant, but it is significantly related to Internet altruism behavior, Internet impression management, and self-esteem.

**Table 1:** Pearson correlations and descriptive statistics for the main study variables.

Variable	M ± SD	gender	CN	IAB	IIM	Self-esteem
gender	1.379 ± 0.486	1				
CN	5.070 ± 1.101	−0.065	1			
IAB	2.550 ± 0.838	−0.084*	0.578**	1		
IIM	3.019 ± 1.097	−0.124**	0.447**	0.652**	1	
Self-esteem	2.934 ± 0.490	0.107**	0.441**	0.267**	0.174**	1

Note. ***p < 0.001, **p < 0.01, *p < 0.05.

IAB: Internet altruistic behavior IIM: Internet Impression Management CN: Communal Narcissism.

### 3.3 Mediation effect analysis

In order to examine the mediating role, a regression analysis using Hayes’ PROCESS Model 4 was conducted. The results indicate that communal narcissism has a significant direct predictive effect on Internet altruistic behavior (β = 0.27, t = 12.08,p < 0.001). After adding Internet impression management as a mediating variable, communal narcissism still significantly predicts Internet altruistic behavior (β = 0.44, t = 18.36,p < 0.001). Therefore, Internet impression management plays a mediating role between communal narcissism and Internet altruistic behavior. Bootstrap tests showed that this mediating effect was significant, with a 95% confidence interval of 0.17 to 0.26 and a mediating effect of 0.22 Table 2.

**Table 2:** Direct and indirect analysis.

Mediation Path	SE	t	95%CI
CN → IAB	0.02	12.08	[0.229,0.317]
CN → IIM	0.03	12.95	[0.378,0.512]
CN → IIM → IAB	0.24	18.36	[0.393,0.487]

### 3.4 Moderation effect analyses

The independent sample t-test results revealed significant gender differences in Internet altruistic behavior, Internet impression management, and self-esteem (t = 2.12, df = 673, p < 0.05; t = 3.25, df = 673, p < 0.01; t = −2.80, df = 673, p < 0.05). Consequently, gender will be used as a control variable in the subsequent analysis. The moderated mediation model, with self-esteem as the moderating variable, was tested using structural equation modeling. The data fit the model well2: χ²/df = 3.70, p < 0.05, CFI = 0.99, TLI = 0.98, SRMR = 0.04 (90% CI [0.02, 0.11]). According to Hayes, Zhonglin Wen et al. [66], the moderated mediation analysis (see Table 3) demonstrates that when Internet impression management is included as a mediating variable and self-esteem as a moderating variable in the regression equation, communal narcissism shows a significant positive predictive effect on Internet impression management. Additionally, both communal narcissism and Internet impression management have a significant positive predictive effect on Internet altruistic behavior. The interaction term of communal narcissism and self-esteem has a significant negative predictive effect on Internet impression management (β = −0.186, t = −2.82, p < 0.01) and a significant positive predictive effect on Internet altruistic behavior (β = 0.132, t = 5.27, p < 0.001). This indicates that self-esteem not only moderates the direct prediction of communal narcissism on Internet altruistic behavior but also moderates the predictive effect of communal narcissism on Internet impression management.

**Table 3:** Moderated mediation model.

	Model 1: IAB			Model 2: IIM		
	β	SE	95%CI	β	SE	95%CI
CN	−0.331	0.115	[-0.557, -0.105]	0.981	0.194	[0.600, 1.362]
Self-esteem	−1.013	0.208	[-1.422, -0.064]	0.943	0.356	[0.245, 1.642]
CN*Self-esteem	0.204	0.039	[0.128, 0.280]	−0.186	0.066	[-0.317, -0.057]
Gender	−0.006	0.046	[-0.096, 0.084]	−0.210	0.078	[-0.363, -0.056]
IIM	0.389	0.023	[-1.422,-0.604]			
Sample	675			675		
R 2	0.547			0.218		
F �	F(4,670)=161.35, p = 0.000			F (4,670)=46.694, p = 0.000		

Note. ***p < 0.001, **p < 0.01,*p < 0.05.

IAB: Internet altruistic behavior IIM: Internet Impression Management CN: Communal Narcissism

### 3.5 Simple slope test

**Fig 2:**
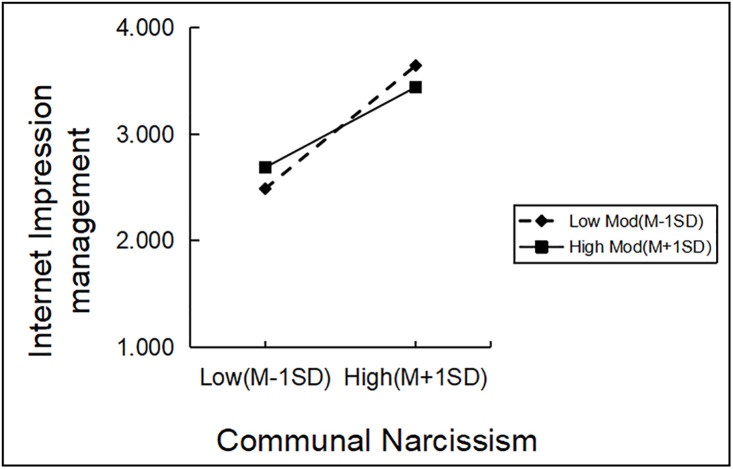
The moderating role of self-esteem in the relationship between communal narcissism and internet impression management.

**Fig 3:**
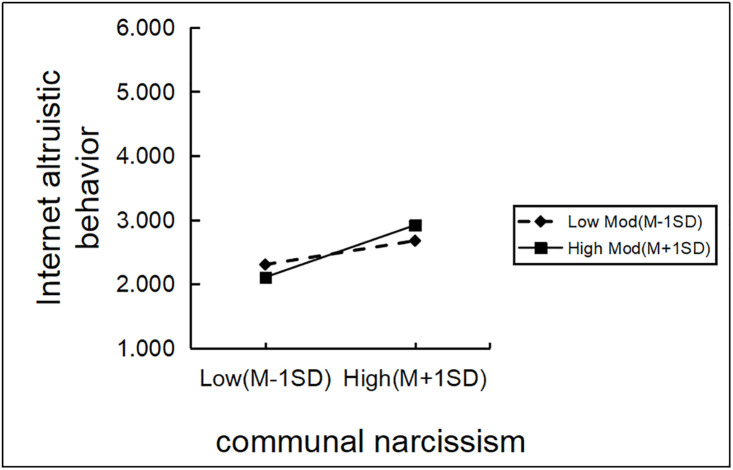
The moderating role of self-esteem in the relationship between communal narcissism and internet altruistic behavior.

In order to further understand the essence of moderation, this study also conducted a simple slope test and drew a simple effect analysis graph (Fig 2,Fig 3). As shown in Fig 2, communal narcissism had a stronger predictive effect on Internet impression management at low levels of self-esteem(B_simple_ = 0.20, t = 11.00, p < 0.001, 95%CI[0.43,0.62])than at high levels of self-esteem(B_simple_ = 0.13, t = 6.50, p < 0.001, 95%CI[0.24,0.45]), cindicating that the moderating role of self-esteem is more pronounced under conditions of low self-esteem. From Fig 3, it can be observed that compared to low levels of self-esteem (β = 0.17, t = 5.58, p < 0.001, 95%CI [0.11,0.23]), communal narcissism more strongly predicted Internet altruistic behavior at high levels of self-esteem (β = 0.37, t = 11.65, p < 0.001, 95%CI[0.31,0.43]).

Finally, this study used the Bootstrap method to analyze the mediating effect of Internet impression management on the relationship between communal narcissism and Internet altruistic behavior at different levels of self-esteem. As shown in Table 5, the results indicate that under conditions of low self-esteem, communal narcissism has a significant mediating effect on Internet altruistic behavior through Internet impression management. Similarly, under conditions of high self-esteem, communal narcissism has a significant mediating effect on Internet altruistic behavior through Internet impression management (See Table 4). Furthermore, the mediating effect under high self-esteem conditions is higher than that under high self-esteem conditions Table 5.

**Table 4:** The moderation effect of self-esteem in the mediation effect test.

Values of self-esteem	B	SE	95%CI
Low self-esteem(M-SD)	0.20***	0.02	[0.16,0.25]
Moderate self-esteem(M)	0.17***	0.02	[0.13,0.21]
High self-esteem(M + SD)	0.13***	0.03	[0.09,0.19]
contrast	−0.07	0.03	[-0.13,-0.02]

Note. ***p < 0.001, **p < 0.01, *p < 0.05.

**Table 5:** The moderation effect of self-esteem.

Values of self-esteem	B	SE	95%CI
Low self-esteem(M-SD)	0.17***	0.03	[0.11,0.23]
Moderate self-esteem(M)	0.27***	0.02	[0.22,0.32]
High self-esteem(M + SD)	0.37***	0.03	[0.31,0.43]

Note. ***p < 0.001, **p < 0.01, *p < 0.05.

## 4. Discussion

This study employs a moderated mediation model to investigate the connection between communal narcissism and college students Internet altruistic behavior. The results illuminate a positive correlation between communal narcissism and college students Internet altruistic behavior, with Internet impression management serving as a mediating factor in this relationship. Futhermore,self-esteem moderated the first stage of this mediation, where higher self-esteem weakened the positive effect of communal narcissism on Internet

### 4.1 Communal narcissism and internet altruistic behavior

A significant positive correlation exists between communal narcissism and internet altruistic behavior among college students, supporting Hypothesis 1. This finding aligns with previous research [23,69]. One explanation is that although individuals high in communal narcissism rate themselves highly on traits such as altruism, kindness, and warmth, their behavior is primarily driven by a need for external validation and reliance on others to maintain their identity [70]. They consistently seek admiration to construct or preserve an idealized self-image [71].While existing research has predominantly focused on individualistic cultural contexts, this mechanism may carry deeper social significance within collectivist cultural settings. For individuals high in communal narcissism in collectivist cultures, internet altruistic behavior serves not only as a strategy for self-presentation but also as an adaptive behavior that aligns with cultural expectations, effectively gaining social recognition and enhancing their reputation within the group. The affordances of online commenting and self-presentation make social media platforms particularly ideal for narcissists to engage in self-display [72] and self-enhancement [73]. Through online spaces, communal narcissists can attract greater attention, thereby reinforcing their self-image and fulfilling intrinsic psychological needs [71].

Furthermore, the altruistic behavior of communal narcissists may reflect not only personal motivations but also conformity to prevailing social and moral norms [74]. They treat social relationships as tools for self-regulation [46], expanding their follower base, gaining attention and recognition, improving others’ evaluations of them, and thereby extending their social networks through actions such as posting photos and short videos across multiple platforms [75]. This series of behaviors aligns particularly well with the cultural logic of collectivist societies, which emphasizes relational harmony, face maintenance, and social evaluation, thereby reinforcing the frequency and visibility of their internet altruistic behavior.

### 4.2 The mediating role of internet impression management

The findings support Hypothesis 2 by confirming that Internet impression management mediates the relationship between communal narcissism and online altruistic behavior among college students. Consistent with prior research, individuals high in communal narcissism often report elevated self-esteem and perceive themselves as exceptionally trustworthy and likable friends [76,77]. The online environment offers a particularly suitable platform for such individuals to strategically curate and display an idealized self-image aimed at garnering social approval [78,74].

Compared to offline interactions, online platforms afford greater flexibility and control over self-presentation, allowing users to selectively highlight desirable traits and construct a positive digital persona [79]. This capacity for curated self-expression reinforces continued social media engagement, as positive feedback further motivates impression management efforts [80]. Within this context, communally narcissistic individuals are particularly inclined to perform online prosocial or altruistic acts—not solely out of genuine concern for others, but as a deliberate strategy to cultivate and maintain a publicly admirable image [74,81]. Indeed, research indicates that those high in communal narcissism spend more time on social media, maintain larger online networks, and invest considerable effort in relationship maintenance—behaviors aligned with strategic self-presentation and image enhancement [82,83].

Notably, this mediated pathway may be especially prominent in collectivist cultural settings such as China, where social harmony, interdependence, and moral reputation are highly valued. In such contexts, altruism is strongly endorsed as a virtuous social behavior, which may further incentivize communally narcissistic individuals to adopt the “altruist” role as a form of strategic self-packaging. Consequently, the link between impression management and altruistic behavior in online spaces is likely more pronounced in collectivist cultures than in individualistic ones.

### 4.3 The moderation of self-esteem

The research findings indicate that self-esteem serves as a significant moderator in the relationships between communal narcissism and Internet impression management and also moderate the relationship between communal narcissism and Internet altruistic behavior. Specifically, for individuals with high communal narcissism, those with lower self-esteem reported engaging in significantly more internet impression management behaviors. Conversely, high communal narcissism predicted greater Internet altruistic behavior primarily among individuals with higher self-esteem.

These results refine and extend prior literature, which has often presented inconsistent patterns regarding narcissism, self-esteem, and social behavior [84]. The observed amplification effect of low self-esteem on the association between communal narcissism and strategic self-presentation partially aligns with the psychodynamic “mask model” [61], suggesting that narcissistic expressions may conceal underlying self-esteem vulnerability. In the online context, individuals with communal narcissistic traits but low self-esteem appear to rely heavily on impression management to construct and regulate a desired social image—a compensatory mechanism aimed at stabilizing a fragile self-concept through curated external feedback.

Conversely, the moderating role of self-esteem in the link between communal narcissism and online altruism reveals a more culturally nuanced pathway. While some studies in individualistic settings associate narcissism with self-promotion rather than genuine prosociality [85,86], the current findings—situated within a collectivist cultural context—suggest that high self-esteem may channel communal narcissism toward outwardly benevolent actions. Specifically, individuals who possess both high communal narcissism and high self-esteem are likely motivated by a need for social recognition, which in collectivist environments is effectively fulfilled through visible prosocial conduct [87]. Supported by greater psychological resources and self-efficacy [88], these individuals engage in online altruism not merely as self-enhancement but as a culturally sanctioned form of self-expression that also reinforces their positive self-view [87].

Thus, self-esteem does not uniformly strengthen or weaken the behavioral outcomes of communal narcissism; rather, it qualitatively shapes its expression. For those with low self-esteem, communal narcissism manifests as image-focused compensation, driven by defensive self-protection. For those with high self-esteem, it facilitates action-oriented expansion, motivated by agentic self-expression and sustained through socially validated altruism. This distinction advances a more integrated understanding of how narcissistic traits interact with self-evaluation to produce divergent online social behaviors.

## 5 Conclusion

In summary, this study investigates the relationship between communal narcissism and college students’ Internet altruistic behavior. Communal narcissism significantly positively predicts college students’ Internet altruistic behavior. Internet impression management mediates the relationship between communal narcissism and college students’ Internet altruistic behavior. Moreover, self-esteem could moderate the relationship between communal narcissism and Internet altruistic behavior and moderate the relationship between communal narcissism and Internet impression management.

## 6 Limitations and implications

There are multiple constraints in this research. Initially, the utilization of a cross-sectional questionnaire design in this study prohibits the strict determination of the causal relationship between variables. Future research could utilize a longitudinal questionnaire design to thoroughly validate the long-term impact of communal narcissism, Internet impression management, and self-esteem on college students’ Internet altruistic behavior. Secondly, this study primarily explored the impact of communal narcissism on college students’ Internet altruistic behavior, without investigating other dimensions of narcissism. Future research can further explore the interaction between other aspects of narcissism and college students’ Internet altruistic behavior. Thirdly, previous studies have found differences in Internet altruistic behavior between different genders, and in the future, further exploration of gender differences can be conducted.

Despite some limitations, this study still holds significant theoretical importance. Firstly, it conducted a more thorough and comprehensive analysis of the relationship between variables by using questionnaire reports. Secondly, further expanding and enriching the research on altruistic behavior, previous studies have focused more on offline altruistic behavior. This study, for the first time, explores the influence of communal narcissism on the Internet altruistic behavior of Chinese university students. Finally, this study is beneficial for better intervening in the Internet altruistic behavior of college students and for providing better guidance to individuals with communal narcissism, prompting them to exhibit more Internet altruistic behavior.

## Supporting information

S1 File Data. (XLSX)
